# Squamous cell carcinoma of the lung presenting as a fungating ulcerated skin lesion: a case report

**DOI:** 10.1186/s13256-022-03352-4

**Published:** 2022-04-26

**Authors:** Rose Chisenga, Tasneem Adenwala, William Kim, Daniel Mujuni, Rachel Samuel

**Affiliations:** Department of Internal Medicine, University of Illinois College of Medicine at Peoria, Peoria, IL USA

**Keywords:** Lung cancer, Squamous cell carcinoma, Skin metastases, Fungating Ulcerated skin lesion, Case report

## Abstract

**Background:**

Our patient presents with a novel presentation of a fungated ulcerated skin lesion as the initial presentation of lung cancer. The literature describes skin metastases from lung cancer as nodular, papular, and zosteriform. Our case is a fungating ulcerated skin lesion which is not widely reported in literature. There is a still a need for more data on the clinical presentation and prognosis of such cases as it will elucidate the diagnostic challenges and treatment management.

**Case presentation:**

We present a case of a 55 year old Caucasian male with a 60-pack-year smoking history initially presenting with a nodule on his right upper back that quickly fungated and ulcerated requiring surgical excision. Biopsy of both the skin lesion and the lung mass confirmed squamous cell carcinoma (SCC) and the lung mass being the primary tumor. The patient’s clinical and functional status severely declined during his hospital stay and was later discharged to hospice without therapeutic intervention. He later expired a month after hospice stay.

**Conclusions:**

Although uncommon, this case clearly illustrates that skin metastases can be the initial finding of primary lung cancer and that not all patients with lung cancer will present with bronchopulmonary symptoms. It also illustrates that a fungating ulcerated lesion can be the initial presentation of lung cancer in addition to nodular, papular, and zosteriform presentations noted in the literature.

## Introduction

Lung cancer is the leading cause of cancer deaths in men and women in the United States accounting for 25% of cancer deaths in 2018 [1, 2]. The most common metastatic sites for lung cancer are to the nervous system, bones, liver, respiratory system, and adrenal glands [3]. Cutaneous metastases of lung cancer are rare but have a reported incidence of 1–12% [4]. Skin lesions were the initial manifestation for lung cancer in 19–62% of these cases presenting before or during the lung cancer diagnosis [5, 6]. These skin lesions lack a distinctive presentation. They have been described as nodular, papular, zosteriform, or fungating; although the latter three presentations are less common [4, 7–11]. They can present as singular or multiple lesions, can be painless or painful, and can vary in color [12]. The location of the lesions can metastasize anywhere on the skin surface but are mostly found in the chest, abdomen, and back [4, 5, 12]. Patients with cutaneous metastases from lung cancer have poor prognosis with a mean survival of 5–6 months after the appearance of skin lesions [4, 7, 13, 14].

Although there is growing evidence in the literature characterizing cutaneous manifestations of lung cancer, there is still a need for more data on the clinical presentation and prognosis of such cases. Especially in these uncommon cases where cutaneous manifestations are the initial presentation. It is unclear how to approach a patient who may present initially with a skin lesion as the first clinical presentation for lung cancer, as is the case in our patient. Our case was unique as the patient presented with a fungating skin lesion as the first clinical manifestation as most of these uncommon cases are described as nodular skin lesions [5]. Another atypical finding from our case was that the biopsy showed squamous cell carcinoma of the lung (LSCC) and the same histopathology was discovered in his skin lesion as LSCC is less likely to have cutaneous manifestations [4, 5]. To form a better understanding of the clinical progression and characterization of metastatic skin lesions from lung cancer, we present the case of a 55 year-old male with his history and workup in order to discuss how it relates to the current literature.

## Case presentation

**Fig. 1:**
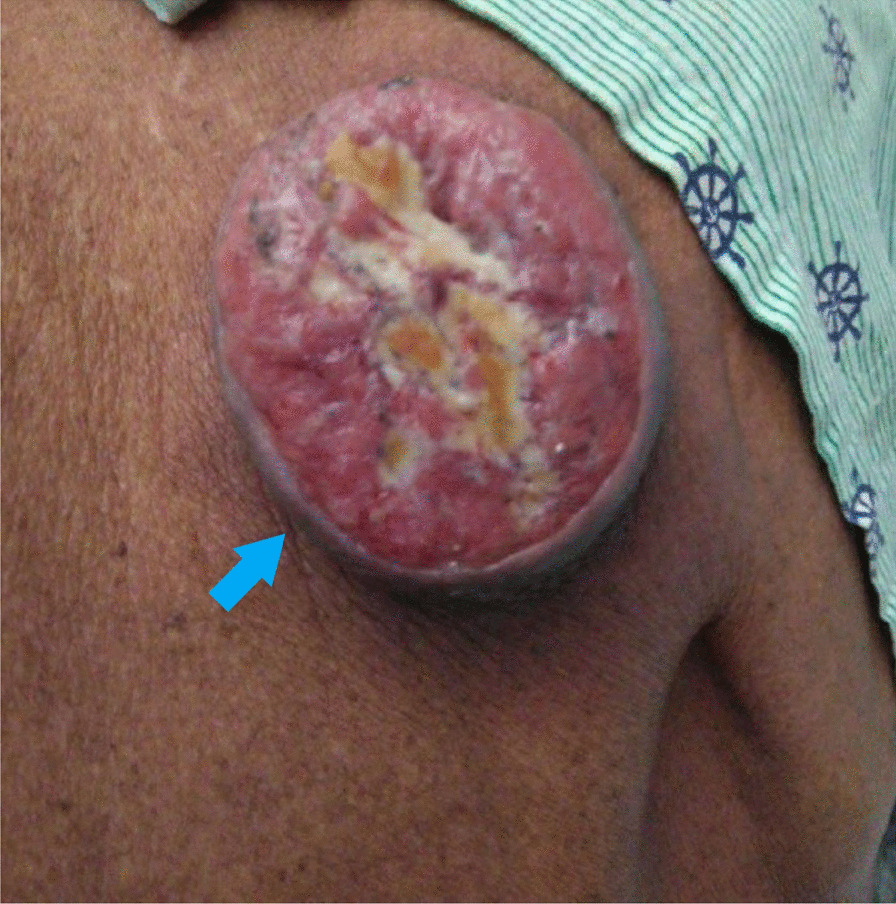
Skin lesion (blue arrow) on the infraspinous region of the right shoulder prior to excision

**Fig. 2:**
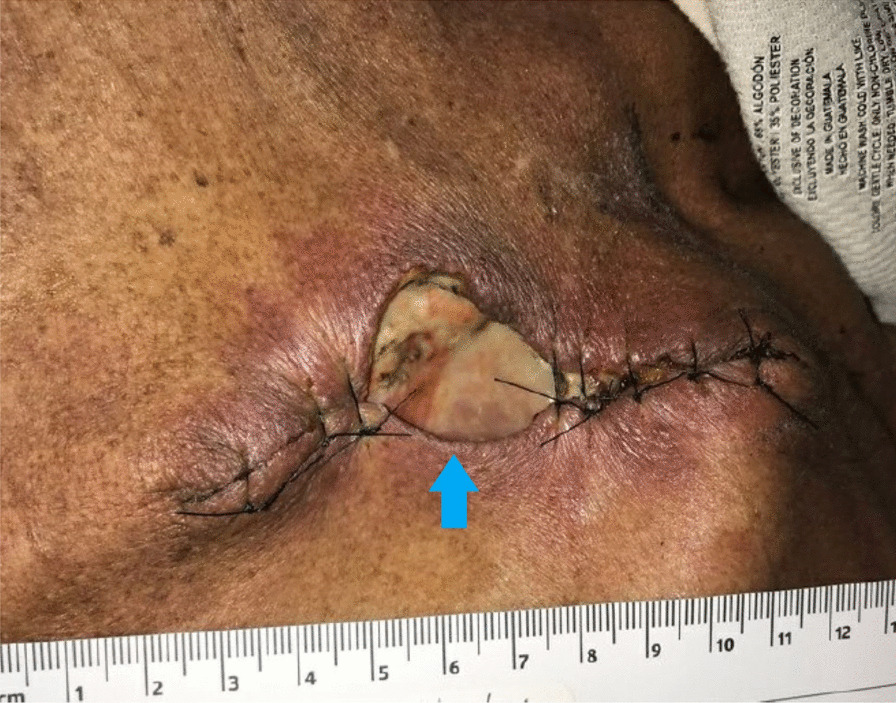
Wound after excision with sutures in place (blue arrow)

**Fig. 3:**
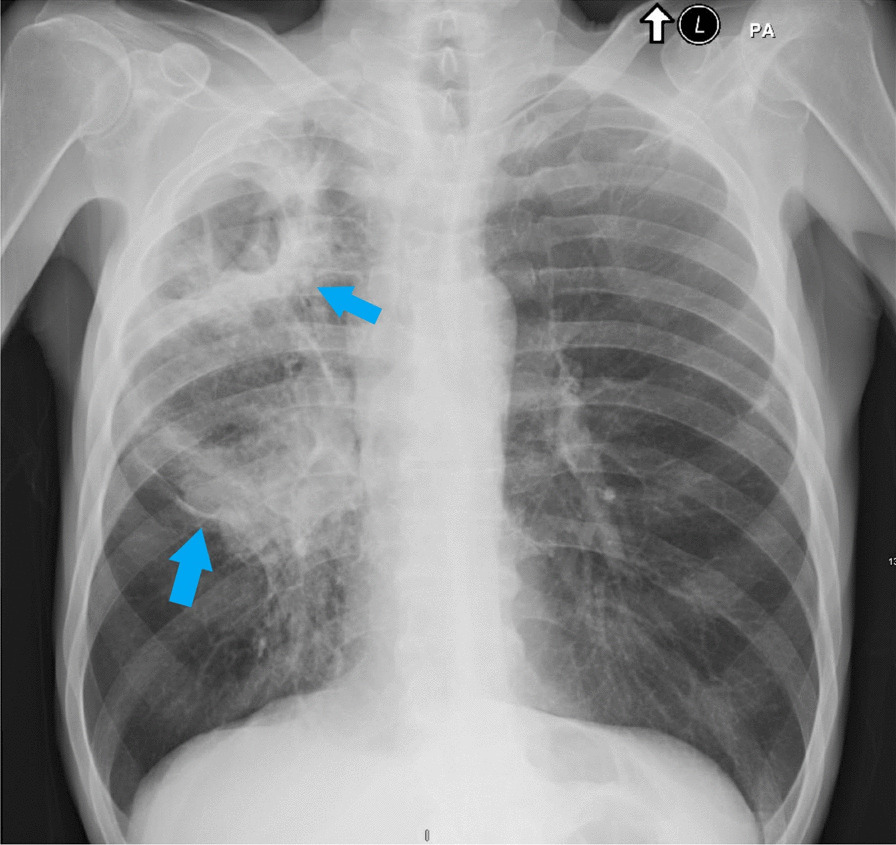
Chest radiograph showing large cavitary lesion in the right upper lung with right hilar mass (blue arrows)

**Fig. 4:**
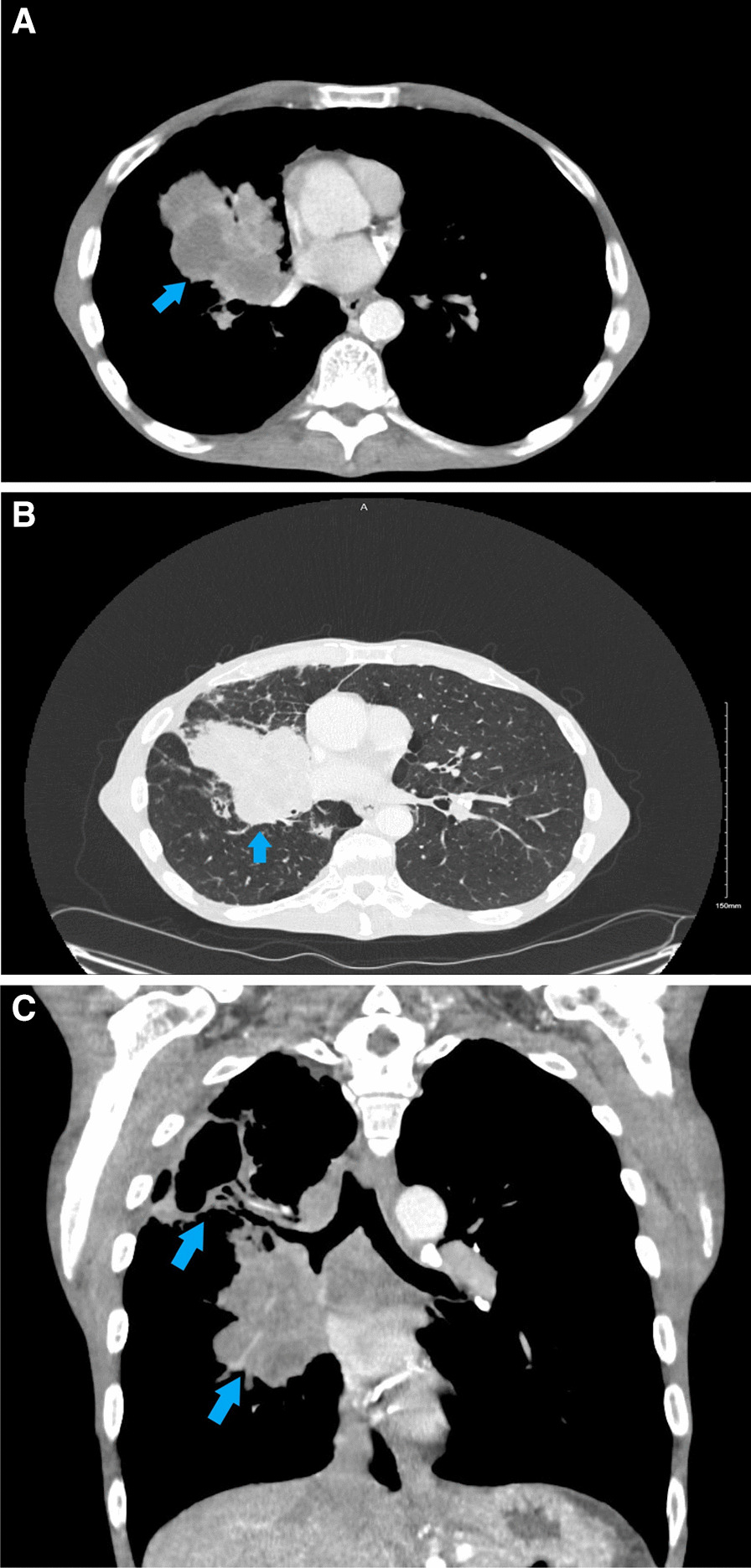
**A** Trans-axial computerized tomography (CT) Chest showing right upper lung mass (blue arrow). **B** Trans-axial computerized tomography (CT) Chest in lung window showing right upper lung mass (blue arrow). **C** Coronal view computerized tomography (CT) chest showing the right upper lobe lung mass (blue arrow). Findings worrisome for extensive malignancy involving the mediastinum, right hilum, and right upper lobe

**Fig. 5:**
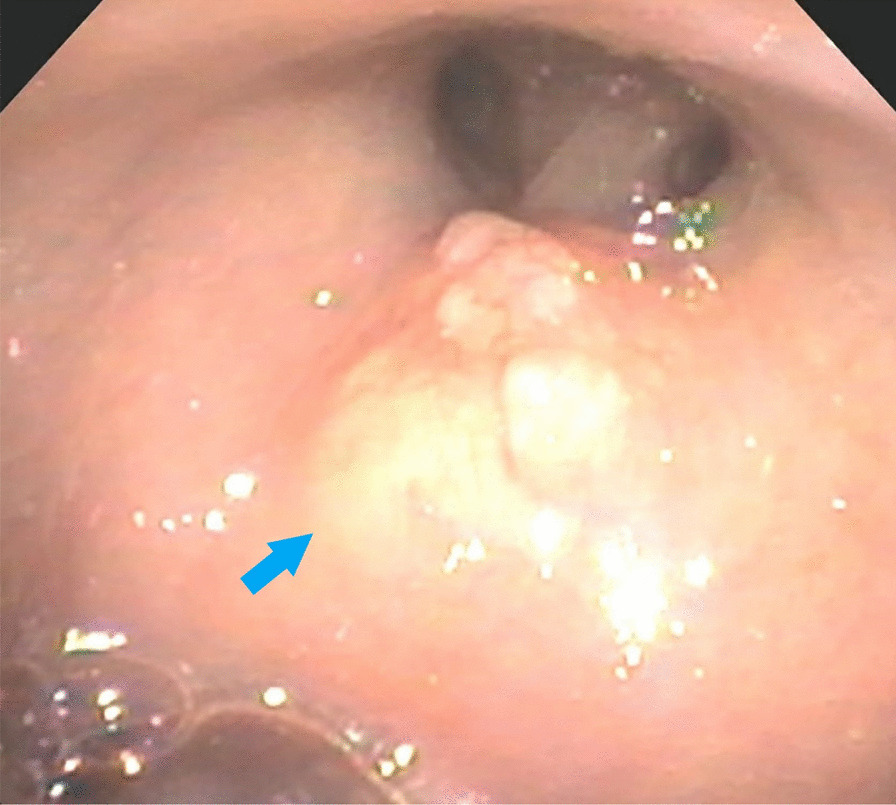
Bronchoscopy shows single small fixed, friable, irregular, nodular and oval lesion (blue arrow) in the right main stem bronchus

A 55 year-old Caucasian male was admitted to the hospital with complaints of worsening generalized weakness. He had a past medical history of tobacco abuse with a 60-pack-year smoking history without any other pertinent medical and family history. At initial presentation, the patient was seen at an urgent care with complaints of a new skin nodule on his right upper back. The patient was referred to dermatology via urgent care, but the patient was lost to follow up. Two months later, it grew and ulcerated, prompting a surgical consult by his PCP for biopsy and excision (Fig. 1). The ulcerated skin lesion became painful and developed purulent drainage requiring antibiotics. During this time, he also noticed weight loss, loss of appetite, and worsening constipation. He denied coughing, hemoptysis, shortness of breath, or any other respiratory symptoms. On physical examination he had normal vital signs and was cachectic. He had a post excision wound on the right upper back measuring about 10 cm in greatest diameter surrounded by mild erythema with sutures in place (Fig. 2). No drainage was noted. He had three palpable, non-tender and freely mobile right posterior cervical lymphadenopathy measuring 2 cm × 2 cm in size. On auscultation of lungs, he had equal air entry bilaterally and was without wheezes or crackles. Otherwise, he had a unremarkable physical exam including normal cardiac exam. Lab work revealed hypercalcemia of 16.3 mg/dL, which was corrected for his albumin. PTHrP was elevated with a low normal PTH. Renal function was normal (Table 1). Chest x-ray revealed a large cavitary lesion in the right upper lung lobe and right hilar adenopathy or a mass (Fig. 3). CT chest with contrast showed extensive malignancy most significantly involving the mediastinum, right hilum, and right upper lobe with areas of interstitial thickening and nodularity in the right upper lobe and right middle lobe and Indeterminate partially exophytic mass in the posterior upper right chest measuring about 3.8 × 6.2 cm (Fig. 4). He underwent bronchoscopy with endobronchial ultrasound which showed a large subcarinal mass and a small fixed, friable, irregular, oval and vascular lesion with less than 25% obstruction in the right main stem (Fig. 5). The histopathology report of the skin lesion was consistent with metastatic poorly differentiated squamous cell carcinoma, mainly invading the dermis and subcutaneous tissue with no dysplastic changes in the epidermis. Smaller nodules were also invading the angiolymphatic vessels. The sections of surgical margins were free of tumor cells. Bronchial washings and FNA of hilar lymph node pathology results confirmed non-small cell carcinoma favoring squamous cell carcinoma. They stained positive for p40 and negative for TTF1. The tumor cells PD-L1 membranous positivity was 5%. Our patient had LSCC and nonkeratinizing SCC in his skin biopsy which made it more likely that it was Stage IV LSCC with metastasis to the skin than a primary cutaneous tumor in addition to the primary pulmonary tumor.

**Table 1:** Laboratory values

Laboratory test	Value	Reference range
White blood cell count	9.99 10 (3)/mcl	4.00–12.00 10 (3)/mcL
Red blood cell count	4.32 10 (3)/mcl	4.40–5.80 10 (6)/mcL
Hematocrit	37.1%	38.0–50.0%
Hemoglobin	11.7 g/dL	13.0–16.5 g/dL
MCV	85.9 fL	82.0–96.0 fL
Platelet count	425 10(3)/mcl	140–440 10(3)/mcL
Neutrophils	59.0%	40.0–68.0 %
Lymphocytes	32.5%	19.0–49.0 %
Monocytes	6.2%	3.0–13.0 %
Eosinophils	1.3%	0.0–8.0 %
Basophils	0.5%	0.0–1.0 %
Absolute neutrophils	5.89 10 (3)/mcl	1.40–5.3 10 (3)/mcL
Absolute lymphocytes	3.25 10 (3)/mcl	0.90–3.30 10 (3)/mcL
Sodium	135 mmol/L	136–145 mmol/L
Potassium	4.3 mmol/L	3.5–5.1 mmol/L
Creatinine	1.14 mg/dL	0.70–1.30 mg/dL
Calcium	15.7 mg/dL	8.7–10.5 mg/dL
Albumin	3.3 g/dL	3.5–5.0 g/dL
PTH	11 pg/mL	13–85 pg/mL
PTH related peptide (6/28/2021)	32 pmol/L	< or = 4.2 pmol/L
White blood cell count	5.55 10 (3)/mcl	4.00–12.00 10 (3)/mcL
Red blood cell count	3.28 10 (3)/mcl	4.40–5.80 10 (6)/mcL
Hematocrit	28.6%	38.0–50.0 %
Hemoglobin	9.3 g/dL	13.0–16.5 g/dL
MCV	87.2 fL	82.0–96.0 fL
Platelet count	316 10 (3)/mcl	140–440 10 (3)/mcL
Neutrophils	60.5%	40.0–68.0 %
Lymphocytes	29.2%	19.0–49.0 %
Monocytes	6.7%	3.0–13.0 %
Eosinophils	3.1%	0.0–8.0 %
Basophils	0.5%	0.0–1.0 %
Absolute neutrophils	3.36 10 (3)/mcl	1.40–5.3 10 (3)/mcL
Absolute lymphocytes	1.62 10 (3)/mcl	0.90–3.30 10 (3)/mcL
Sodium	137 mmol/L	136–145 mmol/L
Potassium	3.1 mmol/L	3.5–5.1 mmol/L
Creatinine	0.6 mg/dL	0.70–1.30 mg/dL
Calcium	8.8 mg/dL	8.7–10.5 mg/dL

*mcl* microliters,* MCV* mean corpuscular volume,* mmol* millimoles per liter,* mg* milligram,* L* liters,* dL* deciliter,* PTH* parathyroid hormone,* pg* picogram,* pmol* picomoles per liter,* fL* femtoliter

**Fig. 6:**
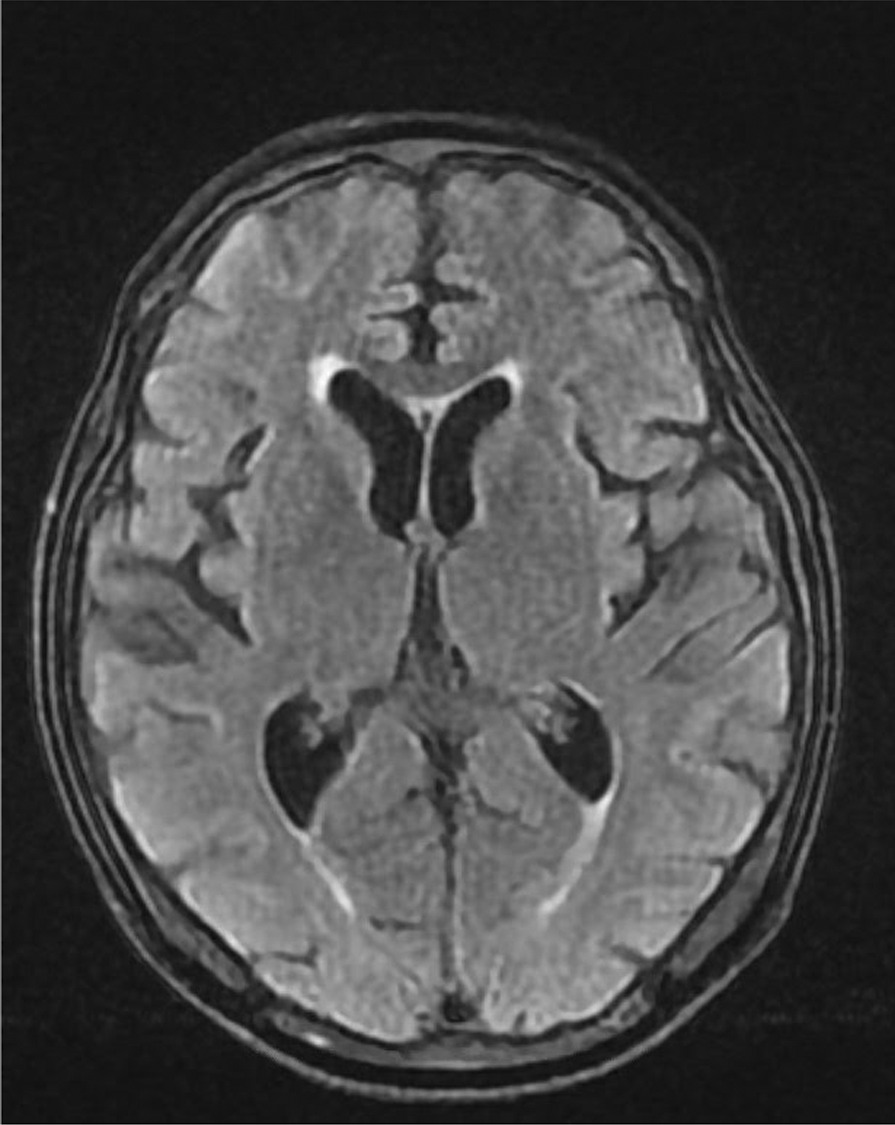
Magnetic resonance imaging (MRI) of the brain combined shows moderate diffuse cerebral and cerebellar atrophy without evidence for intraparenchymal brain metastasis

**Fig. 7:**
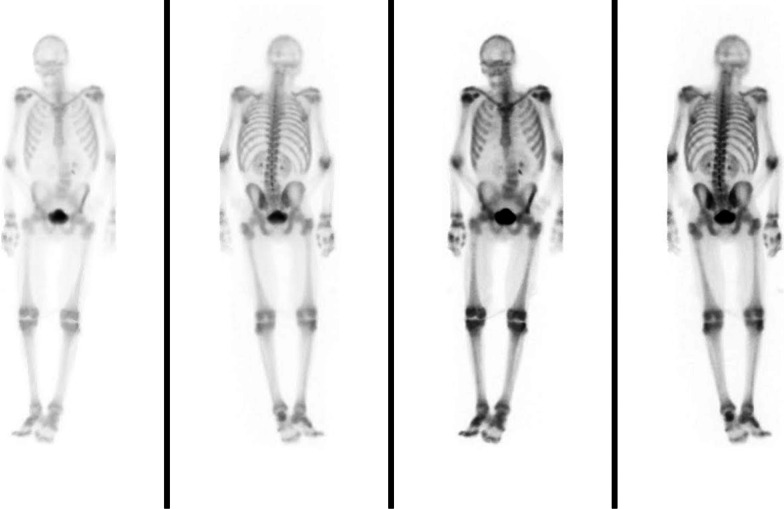
Nuclear medicine (NM) bone scan shows diffuse increased osseous activity without focal suspicious lesion

Further work-up including CT abdomen and pelvis with contrast showed no metastatic disease (Fig. 6). MRI brain showed no metastatic lesion (Fig. 7) and bone scan was only significant for diffuse increased osseous activity without focal suspicious lesion. The hypercalcemia was resolved with intravenous fluid resuscitation and zoledronic acid. Oncology evaluated the patient and planned to start chemotherapy and immunotherapy as outpatient. No therapeutic intervention or further work-up was completed as the patient’s clinical and functional status severely declined during his hospital stay and was later discharged with hospice. His baseline functional status prior to admission was being able to do activities of daily living independently and ambulated on his own. He later expired a month after hospice stay or 4 months after initial presentation.

The most notable diagnostic challenge was the unusual presentation of the skin lesion as the initial presentation for LSCC which delayed the initial pulmonary work-up. Especially since the patient presented with a fungating lesion without pulmonary symptoms which led investigations towards primary dermatologic cancer. The elevated calcium and PTHrP were the first diagnostic clues which started the diagnostic workup for lung cancer. Before the pulmonary workup, it was thought that the patient had a basal or squamous cell carcinoma of the skin which was pending a biopsy report after surgical excision at that time. There were minimal cultural, linguistic, and financial challenges in caring for this patient.

## Discussion

The strengths of our case reports were the unusual skin manifestation of a fungating skin lesion as the first manifestation of lung cancer. The case also laid out the diagnostic challenges in diagnosing lung cancer and how the skin lesion can delay pulmonary work-up due to considering other differential diagnoses including a primary skin cancer. It also further validated many of the evidence found in literature including the region of skin involvement in relation with the pulmonary tumor and clinical outcomes. The main limitation of this case was that it only involved one patient.

Metastatic cutaneous lesions can mimic many other skin pathologies, making early and accurate diagnosis difficult without prompt biopsy and work up. Metastatic skin lesions have been noted to be nodular, papular, zosteriform; although fungating cases have been noted as well but is less common [4, 7–11]. The characteristics of these skin lesions can vary as singular vs. multiple lesions, painless vs. painful, and vary in color [12]. The location of the lesions can be anywhere on the skin but are most frequently occur in the chest, abdomen, and back [4, 5, 12]. In our case report, the skin lesion which presented initially as a benign appearing painless cutaneous singular cystic nodule on the right upper back quickly grew to become a fungating ulcer. It eventually led to the diagnosis of primary lung cancer. The unique and challenging part of this case was that the patient initially presented without bronchopulmonary symptoms and fungating skin lesion which would delay our diagnosis towards a lung pathology. The only prominent history was that of a 60 pack year of smoking in his initial urgent care visit. The patient developed weight loss and fatigue during the outpatient workup. All histopathological types of lung cancer can metastasize to the skin. Adenocarcinoma of the lung (ACC) and large cell carcinoma of the lung (LCC) are the more common lung cancers to metastasize to the skin, while LSCC is less likely to do so [4, 5, 7, 8, 13]. Our patient had LSCC and the same histopathology was discovered in his skin lesion which is a less typical presentation of LSCC.

Most skin metastases tend to be closer to the primary tumor [14]. Most lung cancers will spread to areas above the diaphragm such as the chest, and tumors in the superior lung lobes tend to metastasize to the skin [8]. The exact processes governing lung cancer tropism have not yet been fully elucidated. In our case, the patient’s skin lesion was on his right upper back in the infraspinous region of the scapula while his primary tumor was in the right upper lung lobe.

Skin metastases from lung cancer are usually poorly differentiated, involve the lymphatic vascular system and are restricted to the dermis and subcutaneous tissue with no epidermal dysplasia [15]. Immunohistochemistry for SCC include p40, Tp63 and Ck5/6. While TTF1 is specific, it is less sensitive for adenocarcinoma. The PDL-1 is an immune modulator that promotes immunosuppression by binding to PD-L1 (PDCD1) [16]. Our patient’s PD-L1 expression was 5%. Studies have shown that in patients with over 50% of PD-L1 positivity, they have an improved response to therapy with monoclonal antibodies such as Pemlizumab [17]. The evidence is inconclusive regarding the prognostic significance of PD-L1 expression in both NSCLC and SCLC [18].

Our patient’s skin lesion is an atypical presentation of cutaneous lung metastasis as it was a painful fungating ulcer with purulent drainage prior to excision. The purulent drainage was resolved with an antibiotic regimen. Current literature suggests nodular and painless lesions are more common, although other presentations have also been described [8]. It is necessary for medical providers to have a high index of suspicion for lung cancer in patients who are chronic smokers even without respiratory symptoms. If a patient presents with a chronic smoking history and a suspicious skin lesion, providers should have lung cancer high in their differential diagnoses.

Assuming the ideal case scenario for our patient, where an oncology workup was initiated from the initial presentation of the skin lesion, the chances for a change in prognosis were very minimal. The average survival is about 5 months after diagnosis of skin metastases in lung cancer [14]. Lung cancer patients who present with bronchopulmonary symptoms like cough, wheezing, hemoptysis or shortness of breath have a better prognosis than those presenting with metastatic manifestations [15]. An annual low dose CT scan screening may have benefited this 55 year old patient. In March 2021, USPSTF’s updated guidelines for lung cancer screening and currently recommends annual low dose CT scan in adults aged 50 to 80 years who have a 20 pack-year smoking history and currently smoke or have quit within the past 15 years. This was a change from the previous guidelines which recommended annual screening in adults aged 55 to 80 years old who had a 30 pack-year smoking history. Based on the findings of the National Lung Screening Trial, an annual low dose CT scan has been found to be favorable in detecting earlier stages of adenocarcinoma and squamous cell carcinoma vs. small cell carcinoma- the latter which is very aggressive and have been infrequently detected at early stages [19]. The number needed to screen with low-dose CT to prevent one death from lung cancer is 320 [19]. Our patient had no chest imaging prior to his initial presentation.

## Conclusion

This case highlights how lung cancer can initially present with skin metastases without any respiratory symptoms. Lung cancer with skin metastasis tends to be more aggressive with a higher mortality rate. Biopsy of both the skin lesions and the primary lung mass is key to confirm the diagnosis. Having a high index of suspicion when a patient with smoking history presents with a skin lesion can be helpful in ensuring prompt skin biopsy while looking for a potential primary lung source. Providers need to ensure implementation of the updated USPSTF guidelines for lung cancer screening. Due to the lack of distinctive presentation of skin metastasis from lung cancer, workup for lung cancer should be considered in the presence of any skin lesions in a high-risk patient.

## Data Availability

All data generated or analyzed during this study are included in this published article  [and its supplementary information files].
